# The Role of Probiotics in Managing Glucose Homeostasis in Adults with Prediabetes: A Systematic Review and Meta-Analysis

**DOI:** 10.1155/2024/5996218

**Published:** 2024-03-18

**Authors:** Chao Sun, Qingyin Liu, Xiaona Ye, Ronghua Li, Miaomiao Meng, Xingjun Han

**Affiliations:** ^1^The Second Affiliated Hospital of Shandong University of Traditional Chinese Medicine, Jinan, Shandong, China; ^2^Shandong University of Traditional Chinese Medicine, Jinan, Shandong, China; ^3^Affiliated Hospital of Shandong University of Traditional Chinese Medicine, Jinan, Shandong, China

## Abstract

**Methods:**

The Preferred Reporting Items for Systematic Reviews and Analysis checklist was used. A comprehensive literature search of the PubMed, Embase, and Cochrane Library databases was conducted through August 2022 to assess the impact of probiotics on blood glucose, lipid, and inflammatory markers in adults with prediabetes. Data were pooled using a random effects model and were expressed as standardized mean differences (SMDs) and 95% confidence interval (CI). Heterogeneity was evaluated and quantified as *I*^2^.

**Results:**

Seven publications with a total of 550 patients were included in the meta-analysis. Probiotics were found to significantly reduce the levels of glycosylated hemoglobin (HbA1c) (SMD -0.44; 95% CI -0.84, -0.05; *p* = 0.03; *I*^2^ = 76.13%, *p* < 0.001) and homeostatic model assessment of insulin resistance (HOMA-IR) (SMD -0.27; 95% CI -0.45, -0.09; *p* < 0.001; *I*^2^ = 0.50%, *p* = 0.36) and improve the levels of high-density lipoprotein cholesterol (HDL) (SMD -8.94; 95% CI -14.91, -2.97; *p* = 0.003; *I*^2^ = 80.24%, *p* < 0.001), when compared to the placebo group. However, no significant difference was observed in fasting blood glucose, insulin, total cholesterol, triglycerides, low-density lipoprotein cholesterol, interleukin-6, tumor necrosis factor-*α*, and body mass index. Subgroup analyses showed that probiotics significantly reduced HbA1c in adults with prediabetes in Oceania, intervention duration of ≥3 months, and sample size <30.

**Conclusions:**

Collectively, our meta-analysis revealed that probiotics had a significant impact on reducing the levels of HbA1c and HOMA-IR and improving the level of HDL in adults with prediabetes, which indicated a potential role in regulating blood glucose homeostasis. However, given the limited number of studies included in this analysis and the potential for bias, further large-scale, higher-quality randomized controlled trials are needed to confirm these findings. This trial is registered with CRD42022358379.

## 1. Introduction

Type 2 diabetes (T2DM) is a global health crisis affecting over 10% of the adult population, which has brought huge economic and social burdens on healthcare systems [1]. Prediabetes, characterized by impaired glucose tolerance (IGT) and impaired fasting glucose (IFG), is a high-risk state of diabetes [2]. The American Diabetes Association (ADA) states that as many as 70% of people with prediabetes will eventually develop diabetes within their lifetime [3]. Effective treatment of prediabetes is crucial, as observational studies have linked it to an increased risk of both microvascular and macrovascular diseases [4–6]. However, lifestyle improvement and medication for adult patients with prediabetes have limitations and side effects [7–9]. Therefore, there is an urgent need to identify affordable, efficient, and easily deployable treatment programs to prevent the progression of prediabetes to T2DM.

Currently, probiotics are widely used as a cheap, safe, and convenient treatment for various diseases [10]. The World Health Organization (WHO) provides a definition for probiotics as “live microorganisms that, when given in sufficient amounts, provide a health advantage to the host” [11]. Increasing evidence has suggested that probiotics may regulate blood glucose, improve blood lipids, and control inflammation, playing an important role in the metabolism and disease state of the host [12–15]. However, despite the wide range of beneficial effects of probiotics, the effect of probiotics on prediabetes is not fully understood.

As far as we know, this is the first study to investigate the effects of probiotics on prediabetic adults and explore how probiotics could manage glucose homeostasis by improving blood glucose and lipid metabolism. Furthermore, we investigated the effects of probiotics on inflammatory factors.

## 2. Materials and Methods

### 2.1. Study Protocol

This systematic review and meta-analysis have adhered to the Preferred Reporting Items for Systematic Reviews and Meta-Analyses (PRISMA) statement (Supplementary Material Table S2 PRISMA 2020 checklist) [16]. The protocol for this study was registered with PROSPERO (No. CRD42022358379). Our protocol initially planned also to assess the effectiveness of prebiotics and synbiotics. However, due to the limited number of studies, only the efficacy of probiotics was evaluated.

### 2.2. Inclusion and Exclusion Criteria

The literature we searched had to meet the following criteria: (1) RCTs; (2) written in English; (3) focus on adults ≥ 18 years without diabetes; (4) meet the diagnostic criteria for prediabetes (WHO and ADA) [2, 3]; (5) probiotic is used in their intervention group, placebo is used in their control group; (6) if at least one of the following data were included: glycosylated hemoglobin (HbA1c), fasting blood glucose (FBG), homeostatic model assessment of insulin resistance (HOMA-IR), insulin, triglycerides (TG), total cholesterol (TC), high-density lipoprotein cholesterol (HDL), low-density lipoprotein cholesterol (LDL), interleukin-6 (IL-6), tumor necrosis factor-*α* (TNF-*α*), and body mass index (BMI). Exclusion criteria include the following: (1) non-RCT design, such as study protocols, similar meta-analysis, reviews, case reports, commentaries, or animal trials; (2) data extraction was insufficient; (3) no English article; (4) participants < 18 years.

### 2.3. Data Sources and Search Strategy

PubMed, Embase, and Cochrane Library were searched for relevant literature published through August 2022. The keywords used were as follows: [(probiotics) OR (Lactobacillus) OR (Saccharomyces) OR (Streptococcus thermophilus) OR (Bifidobacterium)] AND [(Glucose Intolerance) OR (Prediabetic State) OR (intermediate hyperglycaemia) OR (impaired fasting glucose) OR (impaired glucose tolerance OR (impaired glucose metabolism)] (Search strategy in Supplementary Table S1). Two investigators (SC and LQY) independently screened the literature and extracted the data. Firstly, duplicate studies were removed. Then, titles and abstracts were screened to exclude studies not meeting the inclusion criteria. Subsequently, the final study was selected by reading comprehensively.

### 2.4. Data Extraction

Two researchers (SC and LQY) independently extracted the data, and their work was subsequently checked by a third researcher (HXJ) for accuracy. We extracted the following data: author, year of publication, country, BMI, administration form, sample sizes, gender, age, details of intervention, intervention duration, and outcome.

### 2.5. Risk of Bias Assessment

The quality of the included studies was assessed according to the Cochrane Handbook [17] by the two reviewers (YXN and LRH). Multiple aspects of potential bias were considered in this systematic review, such as random sequence generation, allocation concealment, participant and researcher blinding, inadequate outcome data, blinding of outcome evaluator, and selective reporting of the studied variables. According to the above specific evaluation criteria, the included studies were categorized as “low risk,” “high risk,” or “unclear risk.” The disagreements were resolved by a third reviewer (HXJ).

### 2.6. Statistical Analyses

Stata software version 17.0 and Rev Man 5.3 were used for statistical analysis, forest plots, and graph of risk of bias. The statistical significance was set at *p* value (*p*) < 0.05. The standard mean difference (SMD) was utilized as a measure of effect size for continuous outcomes, reported along with 95% confidence intervals (CI). The heterogeneity test utilizes the *I*^2^ statistical value. A random-effects method was used to pool effect sizes for heterogeneity and generalizability [18]. Subgroup analyses according to the regional distribution of participants, study duration, and number of participants were conducted to explore the potential sources. Sensitivity analysis was performed by removing studies that caused heterogeneity. The publication bias was evaluated by the visual inspection of asymmetry in the funnel plots and Egger's test for at least 10 studies.

### 2.7. Quality Assessment

We examined the overall certainty of the evidence for all outcomes using the Grading of Recommendations Assessment, Development, and Evaluation (GRADE) framework methodology [19]. We used the GRADE pro software to assess the certainty of evidence. The quality of evidence was classified into four categories according to the corresponding evaluation criteria, including high, moderate, low, and very low [20].

## 3. Results

### 3.1. Search Results

**Figure 1:**
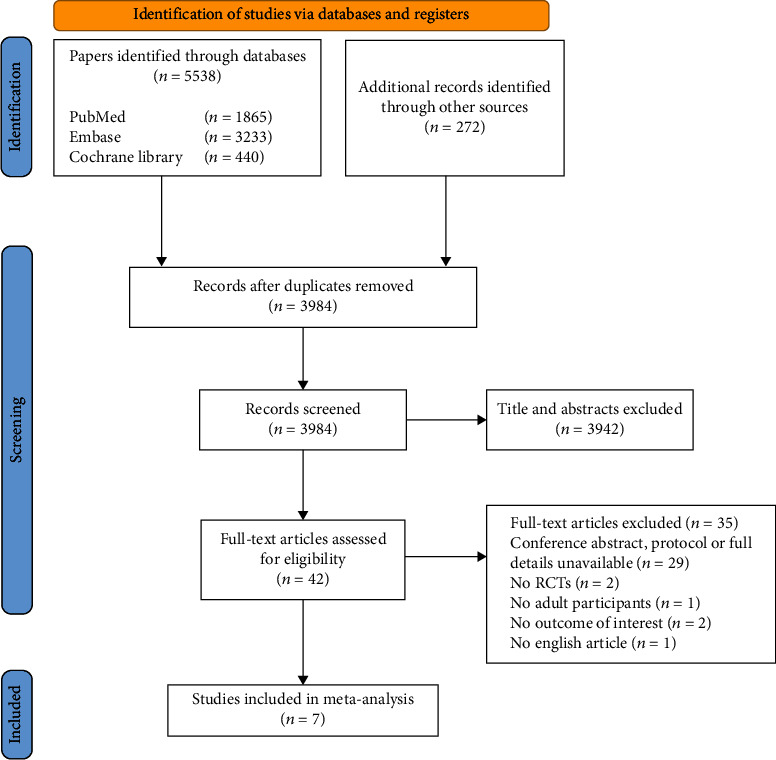
Flow chart for the literature search, study selection, and reasons for exclusion.

A total of 5538 articles were retrieved through literature retrieval, and 272 articles were searched about other relevant systematic reviews and reference lists of the eligible studies. After carefully reviewing the titles, abstracts, duplications, and relevance, we retained 42 articles for further review. A total of 35 articles were subsequently excluded for the reasons listed in Figure 1. In the end, 7 RCTs were included for meta-analysis [21–27].

### 3.2. Study Characteristics

The 7 RCTs were published between 2014 and 2022 and incorporated a total of 550 participants (intervention, 276; control, 274). All included studies were designed as parallel. The included studies were conducted across various geographic locations, specifically with two studies each in New Zealand [21, 25], two studies in Iran [26, 27], two studies in Japan [22, 24], and one study in South Korea [23]. Among them, two articles used composite probiotics [26, 27], while the remaining five articles used single probiotics [21–25]. Four articles were reported to have an intervention time of ≥3 months [21, 24, 25, 27], and three articles had an intervention time of <3 months [22, 23, 26]. The sample size of three articles is ≥30 [21, 22, 24], and that of four articles is <30 [23, 25–27]. The characteristics of 7 RCTs are summarized in Table 1.

### 3.3. Methodological Quality Assessment

**Figure 2:**
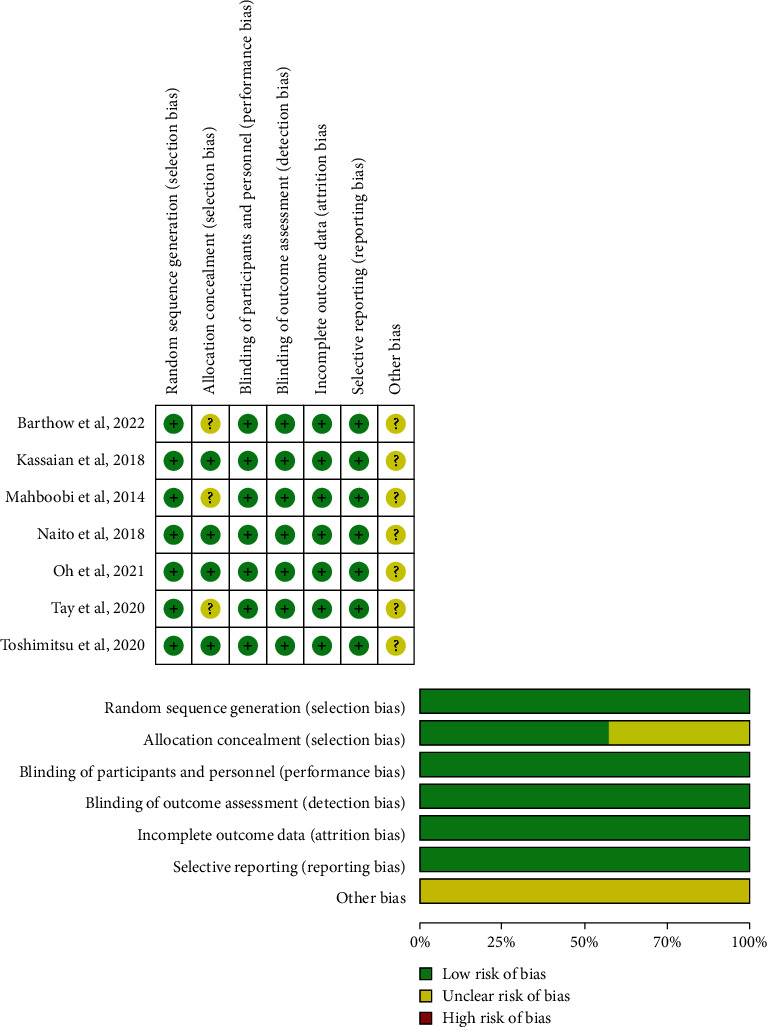
Risk of bias summary.

The summary of the risk of bias is shown in Figure 2. All seven studies described randomization methods, the blindness of participants and/or researchers in the study, and the blindness of outcome evaluation. Outcome data were complete. All seven studies did not have selective reporting. Other obvious sources of bias in the included studies were unknown. However, only four out of seven studies described the use of computer random allocation for allocation concealment.

## 4. Results of Meta-Analyses

### 4.1. Effect of Probiotic Therapy on Blood Glucose

**Figure 3:**
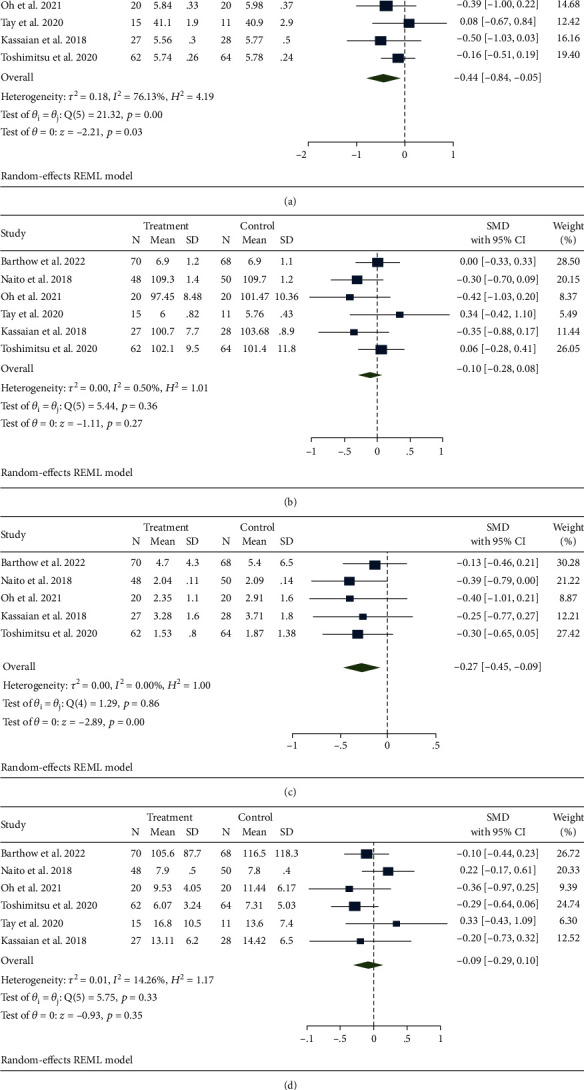
Effects of probiotics on biomarkers of blood glucose. (a) HbA1c; (b) FBG; (c) HOMA-IR; (d) insulin.

The efficacy of probiotics on HbA1c was reported by six studies [21–25, 27]. A significant reduction was observed in most patients who received treatment (SMD, -0.44; 95% CI -0.84, -0.05; *p* = 0.03) with high heterogeneity (*I*^2^ = 76.13%, *p* < 0.001) (Figure 3(a)). We also conducted a subgroup analysis (Table 2). Oceania participants, duration < 3 months and sample size ≥ 30 caused a decrease in heterogeneity. Therefore, we speculated that changes in region, intervention time, and sample size might cause differences in heterogeneity.

A total of 6 studies reported the effects of probiotics on FBG levels [21–25, 27] (Figure 3(b)). No statistically significant difference was observed between the two groups (SMD, -0.10; 95% CI -0.28, 0.08; *p* = 0.27). Slight heterogeneity was found (*I*^2^ = 0.50%, *p* = 0.36). Regarding HOMA-IR, a total of 5 studies mentioned it [21–24, 27] (Figure 3(c)). The probiotic group was prominently more effective than the control group (SMD, -0.27; 95% CI -0.45, -0.09; *p* < 0.001). No heterogeneity was detected between the two groups (*I*^2^ = 0%, *p* = 0.86). The effects of probiotics on insulin were evaluated from six studies [21–25, 27] (Figure 3(d)). No statistically significant difference was observed between the two groups (SMD, -0.09; 95% CI -0.29, 0.10; *p* = 0.35). There was slight heterogeneity (*I*^2^ = 14.26%, *p* = 0.33).

### 4.2. Effect of Probiotic Therapy on Blood Lipids

**Figure 4:**
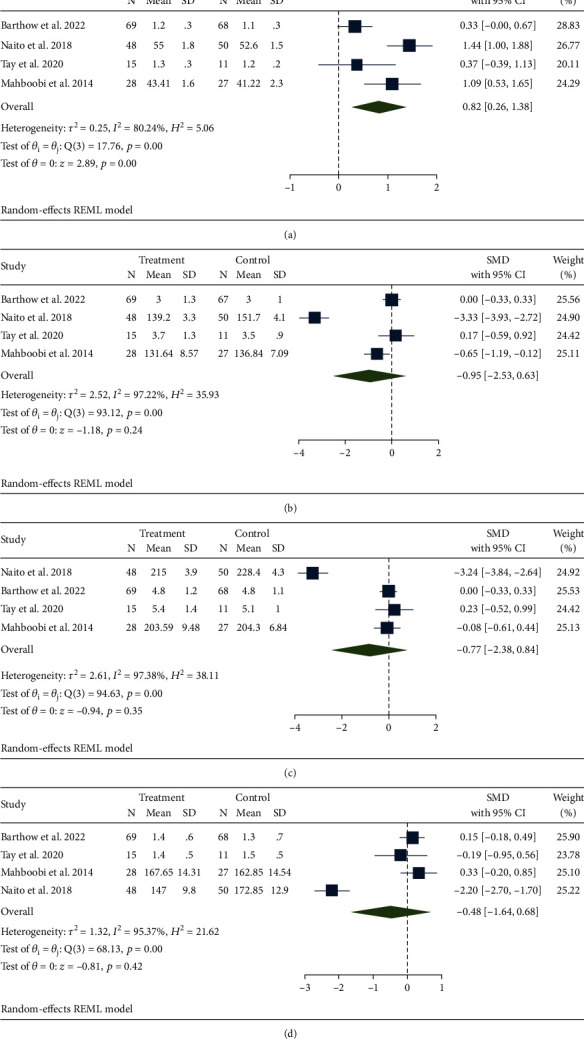
Effects of probiotics on biomarkers of blood lipids. (a) HDL; (b) LDL; (c) TC; (d) TG.

Five studies contained HDL (Figure 4(a)) [21–23, 25, 26]; the probiotics group was significantly more effective than the control group (SMD, 0.82; 95% CI 0.26, 1.38; *p* < 0.001), with a high heterogeneity (*I*^2^ = 80.24%, *p* < 0.001). Four articles examined other blood lipid indicators [21, 22, 25, 26], including LDL (Figure 4(b)), TC (Figure 4(c)), and TG (Figure 4(d)). No significant differences were found between the two groups, and there was a high level of heterogeneity among LDL (SMD, -0.95; 95% CI -2.53, 0.63; *p* = 0.24; *I*^2^ = 97.22%, *p* < 0.001), TC (SMD, -0.77; 95% CI -2.38, 0.84; *p* = 0.35; *I*^2^ = 97.38%, *p* < 0.001), and TG (SMD, -0.77; 95% CI -1.64, 0.68; *p* = 0.35; *I*^2^ = 95.37%, *p* < 0.001).

We conducted a subgroup analysis based on region, intervention time, and sample size (Table 2). The results showed that regional differences were the reason for the high heterogeneity of HDL groups. Region and intervention time were the reasons for the high heterogeneity of the LDL group; region, intervention time, and sample size all contributed to the high heterogeneity of TC and TG groups.

### 4.3. Effect of Probiotic Therapy on Inflammation Factors

**Figure 5:**
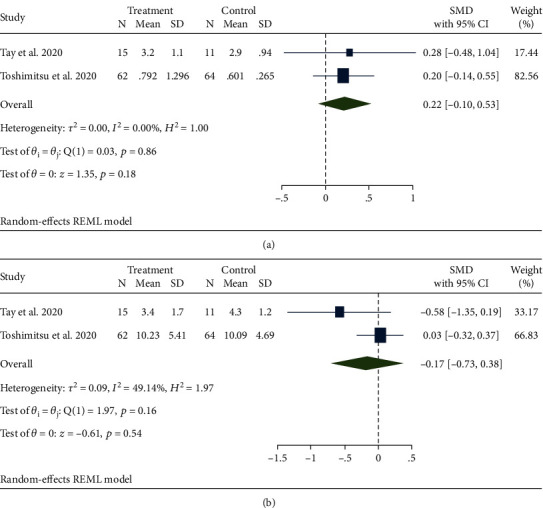
Effects of probiotics on biomarkers of inflammation factors. (a) IL-6. (b) TNF-*α*.

Two studies involved inflammation factors [24, 25], including IL-6 (Figure 5(a)) and TNF-*α* (Figure 5(b)). There was no significant difference between the probiotics and control groups in the groups of IL-6 (SMD, 0.22; 95% CI -0.10, 0.53; *p* = 0.18) and TNF-*α* (SMD, -0.17; 95% CI -0.73, 0.38; *p* = 0.54).

### 4.4. Effect of Probiotic Therapy on Other Indicators

**Figure 6:**
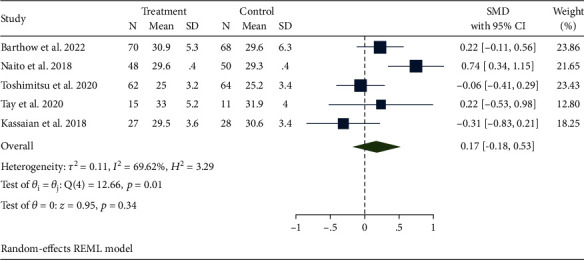
Effects of probiotics on biomarkers of BMI.

Five studies reported BMI [21, 22, 24, 25, 27] (Figure 6). There was heterogeneity between studies (*I*^2^ = 69.62%, *p* = 0.01). The results demonstrate that there was no statistically significant between the two groups (SMD, 0.17; 95% CI -0.18, 0.53; *p* = 0.34). Subgroup analysis indicated that regional differences may be the reason for the high heterogeneity of BMI.

### 4.5. Sensitivity Analysis and Publication Bias

To explore each study's impact on the overall effect size, we omitted each trial from the analysis step by step (Supplementary Material Figure S1-S9). In the case of HOMA-IR, FPG, insulin, LDL, TG, TC, and BMI, there were no significant changes after removing each individual study. After removing the study by Oh et al. [23] (SMD, -0.45; CI, -0.92, 0.02; *p* = 0.061) and Kassaian et al. [27] (SMD, -0.43; 95% CI, -0.91, 0.05; *p* = 0.078), the overall result for HbA1c became statistically significant. In addition, eliminating the study by Mahboobi et al. [26] (SMD, 0.73; CI, -0.01, 1.47; *p* = 0.052), the overall result for HDL was statistically significant. Given that the number of references for each indicator in our research was less than 10, publication bias was not assessed.

### 4.6. Grading of Evidence

The grading of evidence is presented in Table 3. After applying the GRADE framework, we found that the quality of evidence for the effectiveness of FBG, HOMA-IR, and insulin was high. The evidence quality of HbA1c and BMI was moderate. Low-quality evidence was detected for HDL. According to the GRADE protocol [20], evidence regarding TG, TC, and LDL was graded as very low quality.

## 5. Discussion

In this study, we explored the influence of probiotics on the levels of blood glucose, blood lipid, and inflammatory factors in adult prediabetes patients through systematic review and meta-analysis. Our results indicated that probiotics significantly reduced the levels of HbA1c and HOMA-IR and improved the levels of HDL in these patients. There were no significant differences in FBG, insulin, LDL, TC, TG, IL-6, TNF-*α*, and BMI. In addition, subgroup analysis showed that region, intervention time, and sample size might be the reasons for heterogeneity. The GRADE-assessed evidence of our study demonstrated that there were high levels of evidence in the overall analysis of blood glucose indicators, especially in terms of FBG, HOMA-IR, and insulin. However, due to small the number of patients and high heterogeneity, there was low-quality evidence of blood lipid indicators.

Compared to a recent study [28], there are some novelties in our work. Firstly, considering the differences between adolescents and adults, we only explored the effects of probiotics on prediabetes in adults. Stefanaki et al. included a study which involved teenagers [29]. Secondly, studies written in English were included in our work. The study of Yan et al. was Chinese and was removed from our work [30]. Third, our study included the latest RCTs [21, 25]. In addition, we have assessed the overall certainty of evidence across studies based on GRADE guidelines working group. Lastly, we performed comprehensive subgroup analyses to explore the sources of heterogeneity. Compared to the other three articles [31–33], our study has a stronger focus on investigating the effects of probiotics specifically on prediabetes, demonstrating a more targeted approach in this research area. In contrast, the other studies combine both T2DM, prediabetes, and gestational diabetes. Furthermore, our article offers a more comprehensive analysis by encompassing a broader range of prediabetes-related indicators, including blood glucose, lipid profiles, and inflammatory markers. Additionally, our review includes the most extensive collection of articles, highlighting the comprehensiveness of our meta-analysis in the field of prediabetes research.

We found that probiotics could regulate glucose homeostasis, which may be achieved by improving insulin resistance and repairing pancreatic islet *β*-cell function. Firstly, probiotics have been shown to improve insulin resistance by promoting glucagon-like peptide-1 (GLP-1) secretion. GLP-1 is one of the critical mechanisms for improving insulin resistance, primarily by reducing weight and enhancing peripheral tissue sensitivity to insulin [34–36]. When probiotics are consumed, short-chain fatty acids (SCFAs) in the intestine are produced, which combine with the G protein-coupled receptor family 43 (GPR43) and the G protein-coupled receptor family 41 (GPR41). This reaction may increase plasma GLP-1 expression [37]. Some data suggest that the metabolite indole produced by probiotics from tryptophan might also promote GLP-1 secretion by endocrine cells in the intestine [38]. Furthermore, under the influence of probiotics, secondary bile acids activate the G protein bile acid-coupled receptor 5 (TGR5) to stimulate GLP-1 secretion. The findings of this systematic review concluded that probiotics effectively reduced HOMA-IR levels and improved glycosylated hemoglobin levels. At the same time, a recent study has shown that both *Lactobacillus* and *Bifidobacterium* indirectly promote GLP-1 production, which maintains glucose homeostasis [27]. HOMA-IR is an important indicator for evaluating insulin resistance. Probiotics might reduce HOMA-IR by promoting the secretion of GLP-1, which is an important mechanism for reducing glycosylated hemoglobin.

Secondly, the improvement of blood lipid levels by probiotics could also delay the development of insulin resistance. Ingesting probiotics could alleviate the damage to liver tissue cells caused by high cholesterol, leading to a decrease in mRNA expression of crucial enzymes in liver gluconeogenesis and an increase in gene expression associated with glycogen synthase [39, 40]. This could promote liver glycogen formation, reduce liver gluconeogenesis, and increase the body's insulin sensitivity [41]. Related studies have shown that fermented milk from the *Lactobacillus* case *Shirota* strain could improve insulin resistance by reducing blood lipid levels [42]. In our systematic review, we observed that probiotics could improve the levels of HDL without affecting LDL, TG, and TC. We consider that the impact of oral statins on the results has not been excluded during the intervention process. Therefore, we need to further conduct relevant subgroup analysis to ensure the accuracy of the results in the future. Our research has recommended that probiotics could improve HOMA-IR and HDL in managing glucose homeostasis. However, previous studies [28] have shown that probiotics do not have a statistically significant effect on HOMA-IR and HDL. The main reason is the differences in the included articles. The previous studies did not exclude teenagers with prediabetes.

Third, proinflammatory cytokine levels in the blood play a key role in insulin resistance [43]. Inflammatory factors such as IL-6 induce insulin resistance by enhancing serine/threonine phosphorylation of insulin receptor substrate-1 (IRS-1) [44]. Therefore, chronic inflammation is considered an important trigger factor for insulin resistance and leads to elevated levels of glycosylated hemoglobin [45–47]. In our systematic review, it was mentioned that probiotics could prevent hyperglycemia by reducing the levels of IL-6 and TNF-*α*, thereby improving insulin sensitivity [24]. Bacterial lipopolysaccharide (LPS) stimulates the Toll-like receptor 4/nuclear factor kappa B (TLR4/NF-*κ*B) pathway, inducing IL-6, TNF-*α*, and other systemic inflammatory factors [48]. *Botanical murine bacilli* could reduce LPS by inhibiting TLR4/NF-*κ*B expression, which improves the inflammatory state of the body [49, 50]. This systematic review included fewer studies on inflammatory factors and had no significant impact on probiotics in improving inflammatory factors. Therefore, large-scale RCTs with a larger sample size are needed in the future to better understand the effects of probiotics on inflammatory factors.

Additionally, our study noted that probiotics might improve pancreatic islet *β*-cell function. We found that probiotics could enhance pancreatic islet *β*-cell function by increasing the expression of glucose transporter 2 (GLUT2) protein [51]. Previous studies have reported that supplementation with *Bifidobacterial* and *Lactobacillus* could increase the production of *γ*-aminobutyric acid (GABA), which improves pancreatic islet *β*-cell function [52, 53]. Furthermore, probiotics may also inhibit cell apoptosis by upregulating the activity of phosphatidylinositol 3 kinase/phosphorylated serine-threonine protein kinase (PI3K/AKT), which improves pancreatic islet *β*-cell function [54].

Interestingly, our systematic review revealed a finding that probiotics could effectively improve glycosylation. However, they did not have any significant effect on fasting blood glucose levels, which might be attributed to the fact that probiotics mainly regulate postprandial blood glucose. The reason might be that probiotics could reduce blood sugar levels by inhibiting the activity of glucose-producing enzymes in the intestine. The purpose is to reduce the absorption of glucose by the intestine after meals [55]. Therefore, probiotics might be more effective in treating prediabetes patients with IGT.

Several limitations should be considered. Firstly, the number and size of the RCTs included in our study were relatively small. There were variations in the types and dosages of probiotics consumed, as well as the duration of follow-up, which could have influenced our research results. Additionally, only probiotics were utilized as the intervention measure because there were insufficient reports on prebiotics and synbiotics to conduct a meta-analysis. Secondly, in our systematic review, most of the evaluated indicators showed significant heterogeneity, which was likely due to differences in measurement units. Even after using a random effect model and conducting subgroup analysis, we were still unable to eliminate heterogeneity completely. Furthermore, very low to low levels of evidence in our study indicated that probiotics might improve blood lipid levels of adults in prediabetes. Therefore, future long-term observational clinical trials with larger sample sizes and improved quality are crucial to generate more reliable data on the effects of probiotics, prebiotics, and synbiotics. To determine the accuracy of probiotic efficacy, we need to minimize the influence of other drugs on the indicators. Finally, we need to further explain the mechanism of probiotic therapy at the molecular level.

## 6. Conclusions

Our systematic review showed that probiotics clearly have the potential to improve glucose homeostasis. However, to confirm the therapeutic effects of probiotics on adult prediabetes patients, a larger sample size, higher quality, and long-term follow-up RCTs are necessary in the future.

## Figures and Tables

**Table 1:** Characteristics of the included study.

Author, year, country	Administration form	Experiment group					Control group					Outcome	Measurement timepoint (month)
		Sample size	Age (Y) (range or mean ± SD)	Male (%)	BMI (kg/m^2^) (mean ± SD)	Intervention	Sample size	Age (Y) (range or mean ± SD)	Male (%)	BMI (kg/m^2^) (mean ± SD)	Intervention		
Barthow et al., 2022 [21] New Zealand	Capsule	76	39.1-80.3	47.40	31.7 ± 5.6	*Lactobacillus rhamnosus HN001* (6 × 10^9^ cfu)	77	37.5-74.6	57.10	30.2 ± 5	150 mg corn-derived maltodextrin	HbA1c, FBG, HOMA-IR, INS, TG, TC, HDL-C, LDL-C, and BMI	6

Naito et al., 2018 [22] Japan	Milk	48	46.6 ± 7.6	100	29.5 ± 0.4	*Lactobacillus casei YIT* 9029 (>1.0 × 10^11^ cfu)	50	47.4 ± 7.1	100	29.0 ± 0.4	Nonfermented milk	HbA1c, FBG, HOMA-IR, INS, TG, TC, HDL-C, LDL-C, and BMI	2

Oh et al., 2021 [23] Korea	Capsule	20	53.55 ± 10.18	15	25.25 ± 3.14	*Lactobacillus plantarum HAC01* (4 × 10^9^ cfu)	17	56.40 ± 11.57	30	25.03 ± 1.92	Microcrystalline cellulose	HbA1c, FBG, HOMA-IR, INS, TG, TC, HDL-C, and LDL-C	1

Toshimitsu et al., 2020 [24] Japan	Yogurt	62	50.6 ± 6.9	67.74	24.7 ± 3.3	*Lactobacillus plantarum OLL2712* yogurt (≥4 × 10^9^ cfu/112 g of yogurt)	64	51.2 ± 7.6	68.75	24.9 ± 3.2	Placebo yogurt	HbA1c, FBG, HOMA-IR, INS, IL-6, TNF-*α*, and BMI	3

Tay et al.,^∗^ 2020 [25] New Zealand	Capsule	15	52.9 ± 8.7	40	34.7 ± 4.9	*Lactobacillus rhamnosus HN0011* (6 × 10^9^ cfu)	11	54.1 ± 6.4	18	33.6 ± 3.7	Microcrystalline cellulose and dextrose hydrate	HbA1c, FBG, INS, TG, TC, HDL-C, LDL-C, IL-6, TNF-*α*, and BMI	3

Mahboobi et al., 2014 [26] Iran	Capsule	28	51.03 ± 1.37	70.4	28.87 ± 0.80	*Lactobacillus casei* (7 × 10^9^ cfu), *Lactobacillus acidophilus* (1.5 × 10^9^ cfu), *Lactobacillus rhamnosus* (2 × 10^8^ cfu), *Lactobacillus bulgaricus* (2 × 10^8^ cfu), *Bifidobacterium breve* (2 × 10^10^ cfu), *Bifidobacterium longum* (7 × 10^9^ cfu), and *Streptococcus thermophilu* (1.5 × 10^10^ cfu)	27	50.36 ± 1.32	76	29.70 ± 0.80	Starch capsules	TG, TC, HDL-C, and LDL-C	2

Kassaian et al., 2018 [27] Iran	Mixing powder	27	52.9 ± 6.3	48	29.6 ± 3.5	*Lactobacillus acidophilus*, *Bifidobacterium lactis*, *Bifidobacterium bifidum*, and *Bifidobacterium longum* (1 × 10^9^ for each)	28	52.97 ± 5.9	43	30.4 ± 3.2	Maltodextrin	HbA1c, FBG, HOMA-IR, INS, and BMI	6

Outcomes: HbA1c: glycosylated hemoglobin; FBG: fasting blood glucose; HOMA-IR: homeostatic model assessment of insulin resistance; INS: insulin; TG: triglycerides; TC: total cholesterol; HDL-C: high-density lipoprotein cholesterol; LDL-C: low-density lipoprotein cholesterol; IL-6: interleukin-6; TNF-*α*: tumor necrosis factor-*α*; BMI: body mass index. ^∗^Note: both intervention and control groups were under intermittent fasting.

**Table 2:** The results of subgroup analysis.

Biomarkers	Subgroup		Number of studies	Number of participants in experiment/control	SMD (95% CI)	*p* between subgroups	Heterogeneity	
							*I* ^2^ (%)	*p*
HbA1c	Region	Oceania	2 [21, 25]	81/74	-0.19 (-0.50, 0.12)	0.19	0	0.43
		Asia	4 [22-24, 27]	157/162	-0.59 (-1.12, -0.07)		79.82	﹤0.001
	Intervention time^∗^	<3 m	2 [22, 23]	68/70	-0.88 (-1.79, 0.03)	0.17	82.87	0.02
		≥3 m	4 [21, 24, 25, 27]	170/166	-0.23 (-0.44, -0.02)		0	0.61
	Sample size	<30	3 [23, 25, 27]	62/59	-0.34 (-0.69, 0.02)	0.58	0	0.45
		≥30	3 [21, 22, 24]	176/177	-0.56 (-1.28, 0.15)		91.01	﹤0.001

FBG	Region	Oceania	2 [21, 25]	85/79	0.05 (-0.25, 0.36)	0.21	0	0.42
		Asia	4 [22-24, 27]	157/162	-0.20 (-0.45, 0.06)		21.35	0.35
	Intervention time	<3 m	2 [22, 23]	68/70	-0.34 (-0.67, -0.01)	0.10	0	0.77
		≥3 m	4 [21, 24, 25, 27]	174/171	-0.01 (-0.22, 0.20)		0	0.45
	Sample size	<30	3 [23, 25, 27]	62/59	-0.22 (-0.60, 0.16)	0.48	12.05	0.26
		≥30	3 [21, 22, 24]	180/182	-0.06 (-0.26, 0.15)		0	0.35

HOMA-IR	Region	Oceania	1 [21]	70/68	-0.13 (-0.46, 0.21)	0.31	—	—
		Asia	4 [22-24, 27]	157/162	-0.33 (-0.55, -0.11)		0	0.97
	Intervention time	<3 m	2 [22, 23]	68/70	-0.40 (-0.73, -0.06)	0.38	0	0.99
		≥3 m	3 [21, 24, 27]	159/160	-0.22 (-0.43, 0.00)		0	0.78
	Sample size	<30	2 [23, 27]	47/48	-0.31 (-0.71, 0.09)	0.81	0	0.71
		≥30	3 [21, 22, 24]	180/182	-0.26 (-0.46, -0.05)		0	0.58

Insulin	Region	Oceania	2 [21, 25]	85/79	-0.03 (-0.35, 0.30)	0.63	6.18	0.30
		Asia	4 [22-24, 27]	157/162	-0.13 (-0.42, 0.15)		36.26	0.22
	Intervention time	<3 m	2 [22, 23]	68/70	-0.02 (-0.58, 0.54)	0.66	58.71	0.12
		≥3 m	4 [21, 24, 25, 27]	174/171	-0.15 (-0.36, 0.06)		0	0.52
	Sample size	<30	3 [23, 25, 27]	62/59	-0.14 (-0.49, 0.21)	0.77	0	0.33
		≥30	3 [21, 22, 24]	180/182	-0.07 (-0.35, 0.21)		44.39	0.16

TC	Region	Oceania	2 [21, 25]	84/79	0.04 (-0.27, 0.34)	0.28	0	0.58
		Asia	2 [22, 26]	76/77	-1.66 (-4.74, 1.43)		98.34	﹤0.001
	Intervention time	<3 m	2 [22, 26]	76/77	-1.66 (-4.74, 1.43)	0.28	98.34	﹤0.001
		≥3 m	2 [21, 25]	84/79	0.04 (-0.27, 0.34)		0	0.58
	Sample size	<30	2 [25, 26]	43/38	0.02 (-0.41, 0.45)	0.32	0	0.50
		≥30	2 [21, 22]	117/118	-1.61 (-4.78, 1.56)		98.83	﹤0.001

TG	Region	Oceania	2 [21, 25]	84/79	0.10 (-0.21, 0.40)	0.42	0	0.41
		Asia	2 [22, 26]	76/77	-0.94 (-3.42, 1.54)		97.87	﹤0.001
	Intervention time	<3 m	2 [22, 26]	76/77	-0.94 (-3.42, 1.54)	0.42	97.87	﹤0.001
		≥3 m	2 [21, 25]	84/79	0.10 (-0.21, 0.40)		0	0.41
	Sample size	<30	2 [25, 26]	43/38	0.14 (-0.35, 0.63)	0.34	19.12	0.27
		≥30	2 [21, 22]	117/118	-1.02 (-3.33, 1.29)		98.31	﹤0.001

HDL	Region	Oceania	2 [21, 25]	84/79	0.34 (0.03, 0.64)	0.00	0	0.93
		Asia	2 [22, 26]	76/77	1.31 (0.96, 1.65)		0	0.34
	Intervention time	<3 m	2 [22, 26]	76/77	1.31 (0.96, 1.65)	0.00	0	0.34
		≥3 m	22 [21, 25]	84/79	0.34 (0.03, 0.64)		0	0.93
	Sample size	<30	2 [25, 26]	43/38	0.78 (0.07, 1.48)	0.88	55.88	0.13
		≥30	2 [21, 22]	117/118	0.88 (-0.21, 1.96)		93.48	﹤0.001

LDL	Region	Oceania	2 [21, 25]	84/78	0.03 (-0.28, 0.33)	0.14	0	0.69
		Asia	2 [22, 26]	76/77	-1.98 (-4.60, 0.64)		97.61	﹤0.001
	Intervention time	<3 m	2 [22, 26]	76/77	-1.98 (-4.60, 0.64)	0.14	97.61	﹤0.001
		≥3 m	2 [21, 25]	84/78	0.03 (-0.28, 0.33)		0	0.69
	Sample size	<30	2 [25, 26]	43/38	-0.29 (-1.08, 0.51)	0.42	66.77	0.08
		≥30	2 [21, 22]	117/117	-1.65 (-4.91, 1.61)		98.86	﹤0.001

BMI	Region	Oceania	2 [21, 25]	85/79	0.22 (-0.08, 0.53)	0.80	0	1.00
		Asia	3 [22, 24, 27]	137/142	0.14 (-0.48, 0.75)		84.56	﹤0.001
	Intervention time	<3 m	1 [22]	48/50	0.74 (0.34, 1.15)	0.00	—	—
		≥3 m	4 [21, 24, 25, 27]	174/171	0.03 (-0.21, 0.27)		17.57	0.33
	Sample size	<30	2 [25, 27]	42/39	-0.11 (-0.62, 0.39)	0.24	22.95	0.25
		≥30	3 [21, 22, 24]	180/182	0.29 (-0.16, 0.74)		78.61	0.01

^∗^Intervention time: month (m).

**Table 3:** GRADE profile of evidence.

Quality assessment						Summary of findings			Quality of evidence
Outcomes	Risk of bias	Inconsistency	Indirectness	Imprecision	Publication bias	Number of participants in experiment/control	WMD (95% CI)	*I* ^2^ (%)	
HbA1c	No serious limitations	Serious limitation^a^	No serious limitation	No serious limitation	No serious limitation	238/236	-0.44 (-0.84, -0.05)	76.13	⊕ ⊕ ⊕ ◯ Moderate

FBG	No serious limitations	No serious limitations	No serious limitation	No serious limitation	No serious limitation	242/241	-0.10 (-0.28, 0.08)	0.50	⊕ ⊕ ⊕ ⊕ High

HOMA-IR	No serious limitations	No serious limitations	No serious limitation	No serious limitation	No serious limitation	227/230	-0.27 (-0.45, 0.08)	0.00	⊕ ⊕ ⊕ ⊕ High

Insulin	No serious limitations	No serious limitations	No serious limitation	No serious limitation	No serious limitation	242/241	-0.09 (-0.29, 0.10)	14.26	⊕ ⊕ ⊕ ⊕ High

TG	No serious limitations	Very serious limitation^ab^	No serious limitation	Serious limitation^c^	No serious limitation	160/156	-0.84 (-1.64, 0.68)	95.37	⊕ ◯ ◯ ◯ Very low

TC	No serious limitations	Very serious limitation^ab^	No serious limitation	Serious limitation^c^	No serious limitation	160/156	-0.77 (-2.38, 0.84)	97.38	⊕ ◯ ◯ ◯ Very low

HDL-C	No serious limitations	Serious limitation ^ab^	No serious limitation	Serious limitation^c^	No serious limitation	160/156	0.82 (0.26, 1.38)	80.24	⊕ ⊕ ◯ ◯ Low

LDL-C	No serious limitations	Very serious limitation^ab^	No serious limitation	Serious limitation^c^	No serious limitation	160/155	-0.95 (-2.53, 0.63)	97.22	⊕ ◯ ◯ ◯ Very low

BMI	No serious limitations	Serious limitation^a^	No serious limitation	No serious limitation	No serious limitation	222/221	0.17 (-0.18, 0.53)	69.62	⊕ ⊕ ⊕ ◯ Moderate

^a^The test for heterogeneity is significant. ^b^The test for the confidence interval is wide. ^c^The sample size is small. Based on the number of limitations, the quality of outcomes is divided into four categories: high (⊕ ⊕ ⊕ ⊕), moderate (⊕ ⊕ ⊕ ◯), low (⊕ ⊕ ◯ ◯), and very low (⊕ ◯ ◯ ◯) quality.

## Data Availability

The original contributions presented in the study are included in the article/Supplementary Material, and further inquiries can be directed to the corresponding author.

## Supplementary Materials

Supplementary 1 Table S1: search strategy.

Supplementary 2 Table S2: PRISMA 2020 checklist.

Supplementary 3 Figure S1: sensitivity analysis for homeostatic model assessment of insulin resistance (HOMA-IR). Figure S2: sensitivity analysis for glycosylated hemoglobin (HbA1c). Figure S3: sensitivity analysis for fasting blood glucose (FBG). Figure S4: sensitivity analysis for insulin. Figure S5: sensitivity analysis for total cholesterol (TC). Figure S6: sensitivity analysis for triglycerides (TG). Figure S7: sensitivity analysis for high-density lipoprotein cholesterol (HDL-C). Figure S8: sensitivity analysis for low-density lipoprotein cholesterol (LDL-C). Figure S9: sensitivity analysis for body mass index (BMI).
