# Supplementary material for: The Role of Probiotics in Managing Glucose Homeostasis in Adults with Prediabetes: A Systematic Review and Meta-Analysis
Source: J Diabetes Res. 2024 Mar 18;2024:5996218. doi: 10.1155/2024/5996218 (PMC10963111; doi:10.1155/2024/5996218)
Supplement: Supplementary 1 — Table S1: search strategy. [file 5996218.f1.docx]

**Table S1.** Search strategy

| **PubMed** | |
| --- | --- |
| #1 | “Probiotics” [MeSH Terms] OR “Lactobacillus” [MeSH Terms] OR “Saccharomyces” [MeSH Terms] OR “Streptococcus thermophilus” [MeSH Terms] OR “Bifidobacterium” [MeSH Terms] OR “Bacillus subtilis” [MeSH Terms] OR “Enterococcus or Lactococcus”[MeSH Terms] |
| #2 | “probiotic*”[Title/Abstract] OR “lactobacill*”[Title/Abstract] OR “bifido*”[Title/Abstract] OR “bifidu*”[Title/Abstract] OR “lactococc*”[Title/Abstract] OR “saccharomyc*”[Title/Abstract] OR “streptococcus thermophilus”[Title/Abstract] OR “bacillus subtilis”[Title/Abstract] OR “enterococcus faec*”[Title/Abstract] OR “Bulgarian bacillus”[Title/Abstract] |
| #3 | #1 OR #2 |
| #4 | "Glucose Intolerance"[MeSH Terms] OR "Prediabetic State"[MeSH Terms] |
| #5 | “prediabet*”[Title/Abstract] OR “pre diabet*”[Title/Abstract] OR “intermediate hyperglycaemi*”[Title/Abstract] |
| #6 | “impaired fasting glucose”[Title/Abstract] OR “glucose intolerance”[Title/Abstract] |
| #7 | "impaired glucose"[Title/Abstract] AND ("tolerance"[Title/Abstract] OR "metabolism"[Title/Abstract]) |
| #8 | "IFG"[Title/Abstract] OR "impaired fpg"[Title/Abstract] OR "IGT"[Title/Abstract] |
| #9 | ("risk"[Title/Abstract] OR "progress*"[Title/Abstract] OR "prevent*"[Title/Abstract] OR "inciden*"[Title/Abstract] OR "conversion"[Title/Abstract] OR "develop*"[Title/Abstract] OR "delay*"[Title/Abstract]) AND ("diabetes"[Title/Abstract] OR "t2d"[Title/Abstract] OR "NIDDM"[Title/Abstract] OR "type 2"[Title/Abstract] OR "typeII"[Title/Abstract]) |
| #10 | #4 OR#5 OR #6 OR #7 OR #8 OR #9 |
| #11 | #3 AND #10 |
| **Cochrane Library** | |
| #1 | MeSH descriptor: [Probiotics] explode all trees |
| #2 | MeSH descriptor: [Lactobacillus] explode all trees |
| #3 | MeSH descriptor: [Saccharomyces] explode all trees |
| #4 | MeSH descriptor: [Streptococcus thermophilus] explode all trees |
| #5 | MeSH descriptor: [Bifidobacterium] explode all trees |
| #6 | MeSH descriptor: [Bacillus subtilis] explode all trees |
| #7 | MeSH descriptor: [Enterococcus] explode all trees |
| #8 | MeSH descriptor: [Lactococcus] explode all trees |
| #9 | (probiotic* OR lactobacill* OR bifido* or bifidu* OR lactococc* OR saccharomyc* OR streptococcus thermophilus OR bacillus subtilis OR enterococcus faec* OR bulgarian bacillus):ti,ab,kw (Word variations have been searched) |
| #10 | #1 OR #2 OR #3 OR #4 OR #5 OR #6 OR #7 OR #8 OR #9 |
| #11 | MeSH descriptor: [Glucose Intolerance] explode all trees |
| #12 | MeSH descriptor: [Prediabetic State] explode all trees |
| #13 | (prediabet* or pre diabet* or intermediate hyperglycaemi*):ti,ab,kw (Word variations have been searched) |
| #14 | ((impaired fasting glucose) or glucose intolerance):ti,ab,kw (Word variations have been searched) |
| #15 | ((impaired glucose) AND (tolerance or metabolism)):ti,ab,kw (Word variations have been searched) |
| #16 | (IFG or impaired FPG or IGT):ti,ab,kw (Word variations have been searched) |
| #17 | ((risk or progress* or prevent* or inciden* or conversion or develop* or delay*) AND (diabetes or T2D* or NIDDM or "type 2" or "typeII")):ti,ab,kw (Word variations have been searched) |
| #18 | #11 OR #12 OR #13 OR #14 OR #15 OR #16 OR #17 |
| #19 | #10 AND #18 |
| **Embase** | |
| #1 | probiotic agent'/exp OR 'lactobacillus'/exp OR 'saccharomyces'/exp OR 'streptococcus thermophilus'/exp OR 'bifidobacterium'/exp OR 'bacillus subtilis'/exp OR 'enterococcus'/exp OR 'lactococcus garvieae'/exp |
| #2 | probiotic*:ab,ti OR lactobacill*:ab,ti OR bifido*:ab,ti OR bifidu*:ab,ti OR lactococc*:ab,ti OR saccharomyc*:ab,ti OR 'streptococcus thermophilus':ab,ti OR 'bacillus subtilis':ab,ti OR 'enterococcus faec*':ab,ti OR 'bulgarian bacillus':ab,ti |
| #3 | #1 OR #2 |
| #4 | 'glucose intolerance'/exp OR 'impaired glucose tolerance'/exp |
| #5 | prediabet*:ab,ti OR 'pre diabet*':ab,ti OR 'intermediate hyperglycaemi*':ab,ti |
| #6 | 'impaired fasting glucose':ab,ti OR 'glucose intolerance':ab,ti |
| #7 | 'impaired glucose':ab,ti AND (tolerance:ab,ti OR metabolism:ab,ti) |
| #8 | ifg:ab,ti OR 'impaired fpg':ab,ti OR igt:ab,ti |
| #9 | (risk:ab,ti OR progress*:ab,ti OR prevent*:ab,ti OR inciden*:ab,ti OR conversion:ab,ti OR develop*:ab,ti OR delay*:ab,ti) AND (diabetes:ab,ti OR t2d*:ab,ti OR niddm:ab,ti OR 'type 2':ab,ti OR 'typeii':ab,ti) |
| #10 | #4 OR #5 OR #6 OR #7 OR #8 OR #9 |
| #11 | #3 AND #10 |
