# Supplementary material for: The Role of Probiotics in Managing Glucose Homeostasis in Adults with Prediabetes: A Systematic Review and Meta-Analysis
Source: J Diabetes Res. 2024 Mar 18;2024:5996218. doi: 10.1155/2024/5996218 (PMC10963111; doi:10.1155/2024/5996218)
Supplement: Supplementary 3 — Figure S1: sensitivity analysis for homeostatic model assessment of insulin resistance (HOMA-IR). Figure S2: sensitivity analysis for glycosylated hemoglobin (HbA1c). Figure S3: sensitivity analysis for fasting blood glucose (FBG). Figure S4: sensitivity analysis for insulin. Figure S5: sensitivity analysis for total cholesterol (TC). Figure S6: sensitivity analysis for triglycerides (TG). Figure S7: sensitivity analysis for high-density lipoprotein cholesterol (HDL-C). Figure S8: sensitivity analysis for low-density lipoprotein cholesterol (LDL-C). Figure S9: sensitivity analysis for body mass index (BMI). [file 5996218.f3.pdf]

Supplementary Figures

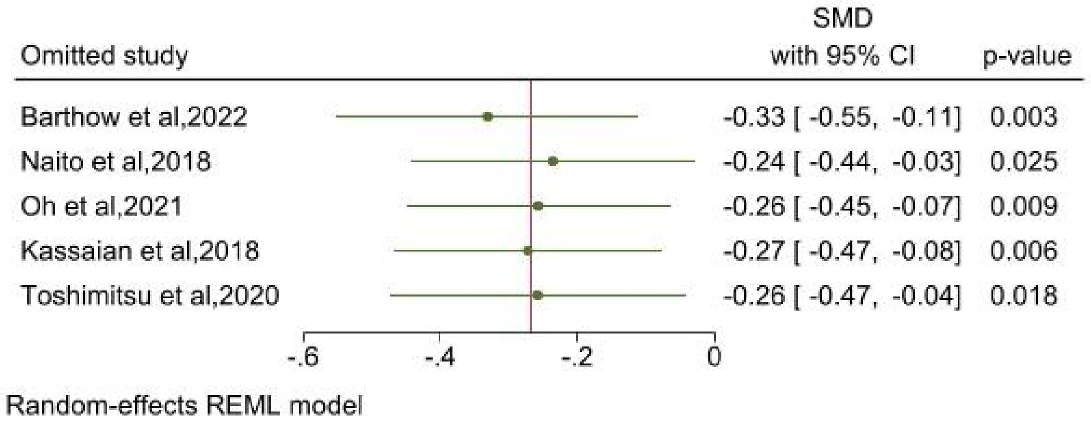

Figure S1 Sensitivity analysis for HOMA-IR

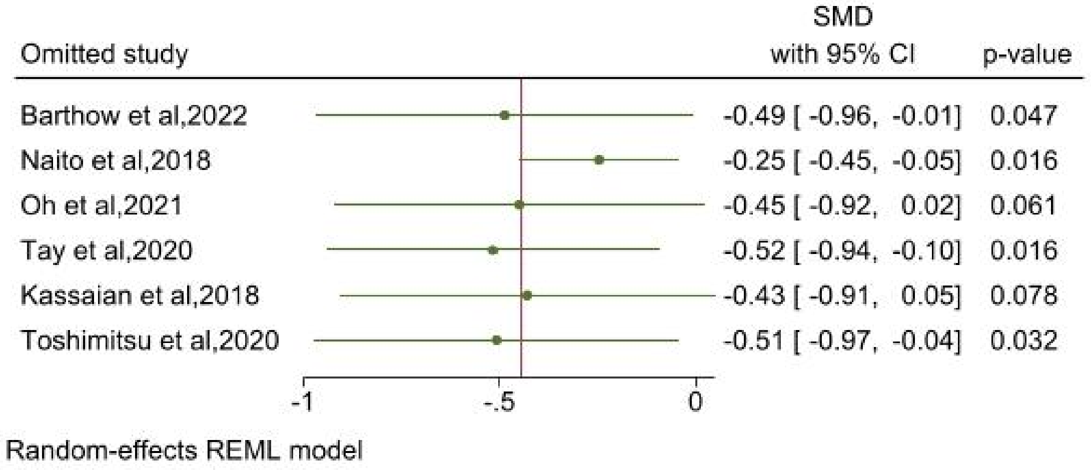

Figure S2 Sensitivity analysis for HbA1c

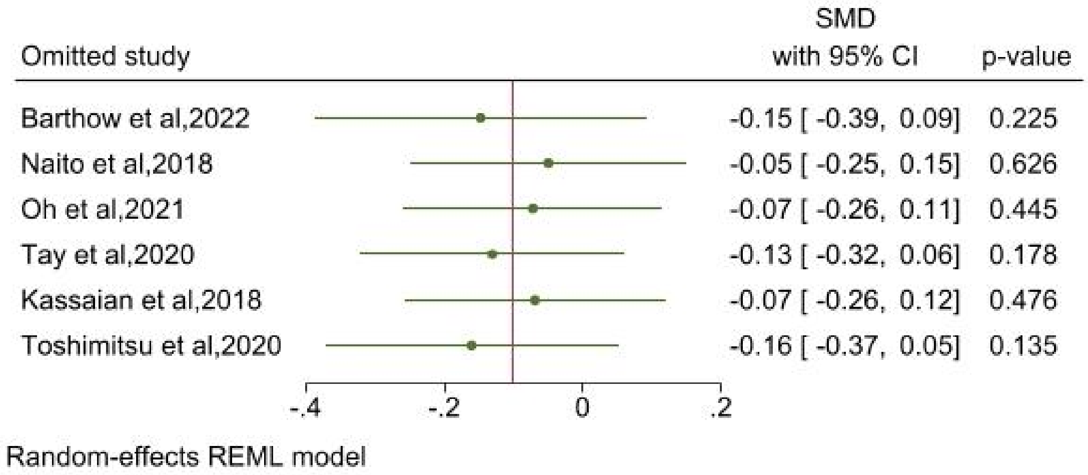

Figure S3 Sensitivity analysis for FBG

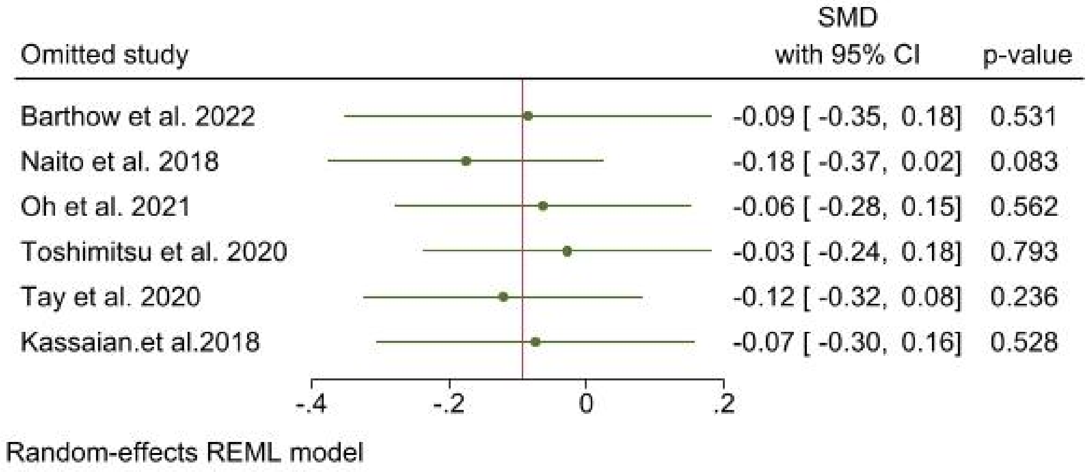

Figure S4 Sensitivity analysis for insulin

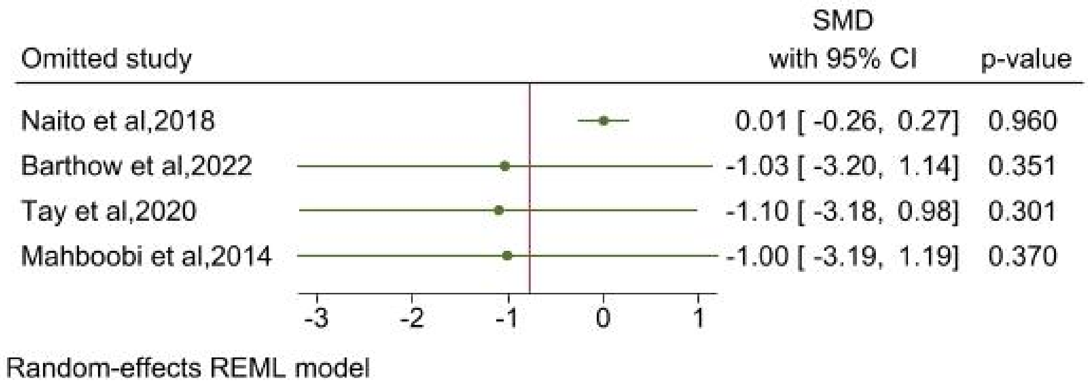

Figure S5 Sensitivity analysis for TC

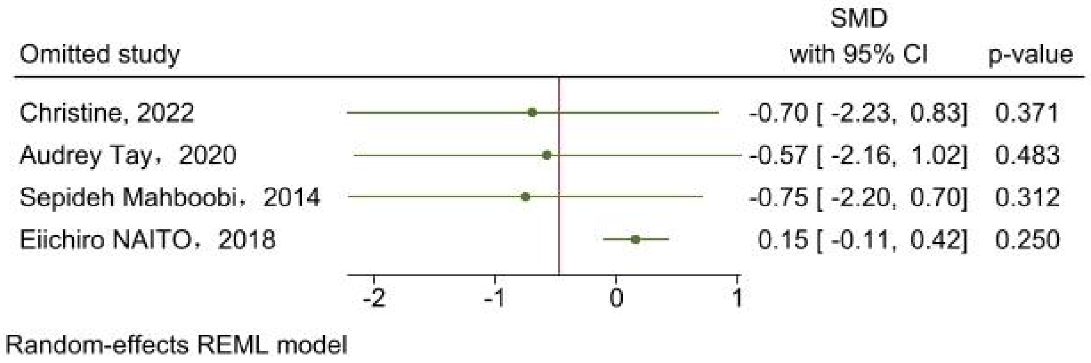

Figure S6 Sensitivity analysis for TG

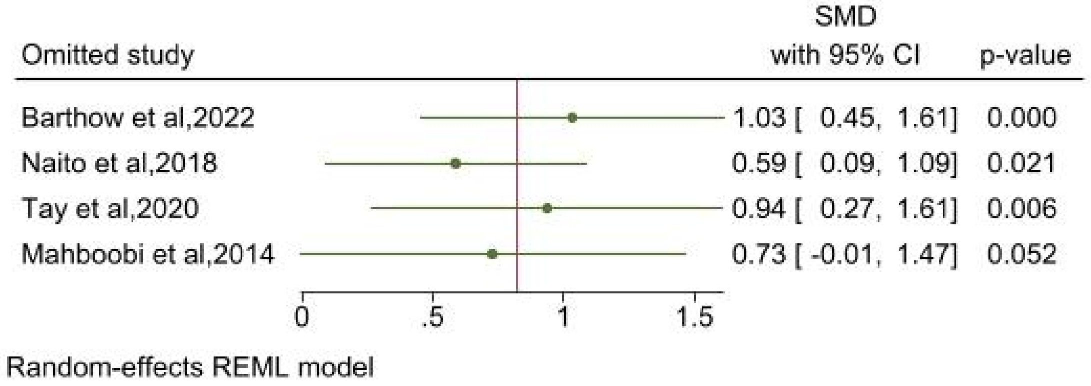

Figure S7 Sensitivity analysis for HDL

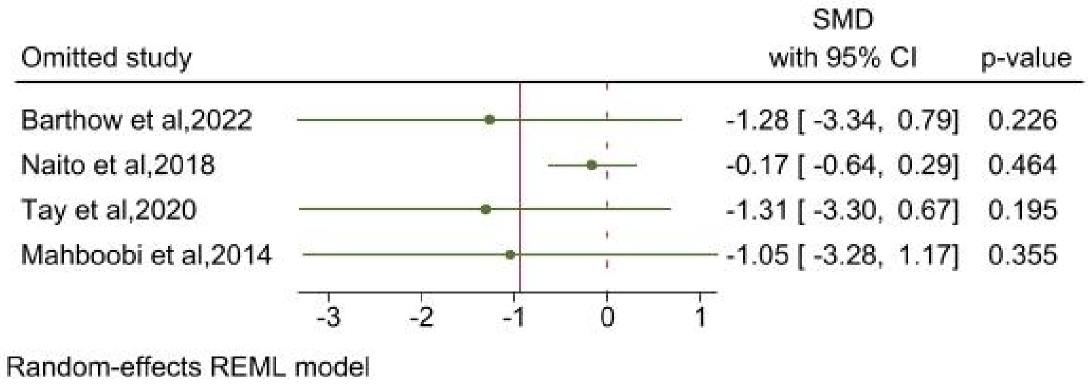

Figure S8 Sensitivity analysis for LDL

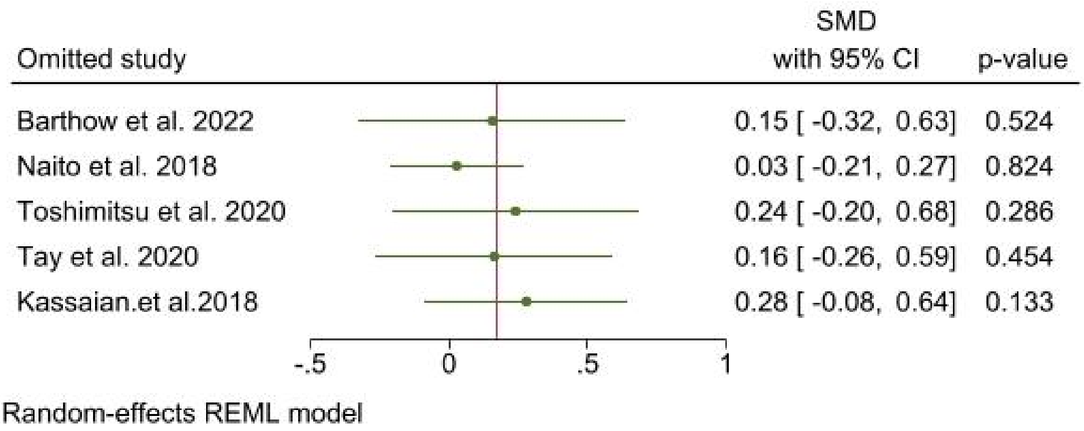

Figure S9 Sensitivity analysis for BMI
